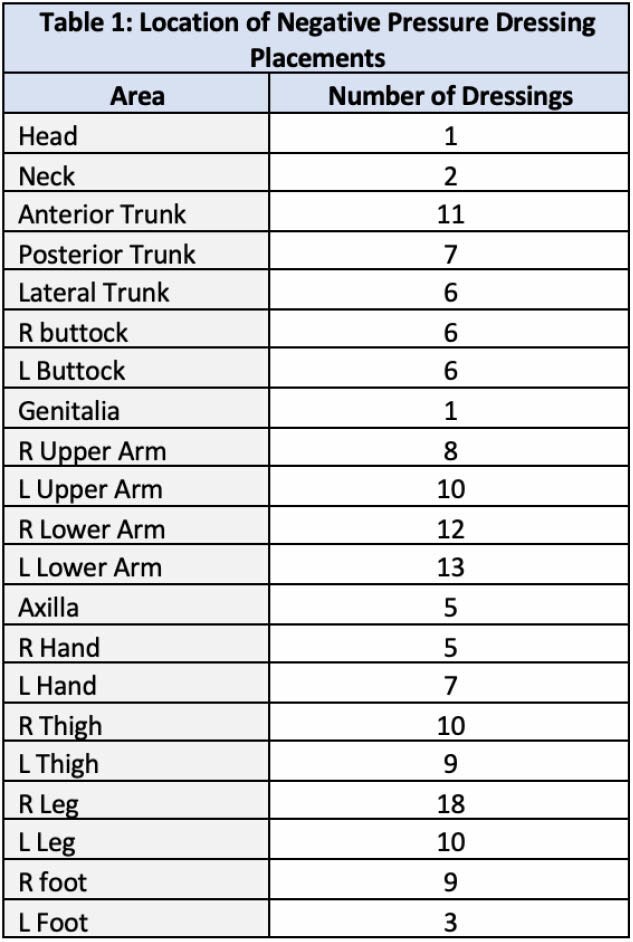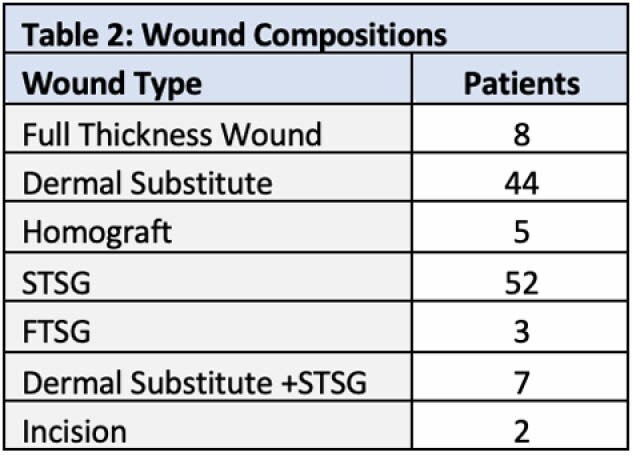# 1003 Physical Therapist Involvement in Negative Pressure Wound Therapy Application in the Inpatient Burn Unit

**DOI:** 10.1093/jbcr/iraf019.534

**Published:** 2025-04-01

**Authors:** Audrey O’Neil, Cassandra Rush, Brett Hartman

**Affiliations:** Richard M. Fairbanks Burn Center; Eskenazi Health; Eskenazi Health

## Abstract

**Introduction:**

Integration of Physical Therapy (PT) into burn wound care has been found to increase therapist productivity, job satisfaction, and multidisciplinary collaboration. However participation of the burn therapist in specific dressing placements has not been examined. PT involvement in dressing application has qualitatively been found to allow early implementation of functional dressings and initiation of contracture prevention. Negative pressure wound therapy (NPWT) specifically has grown in popularity with management of burn wounds due to the ability to protect against shear, manage drainage, and accommodate anatomically complex areas. This study evaluated the impact of PT utilization of NPWT within a single center.

**Methods:**

Retrospective chart review was completed over a 6-month period (March 2024-September 2024) on a 15-bed adult verified burn center. The inpatient unit has 3 dedicated PTs scheduled at 2-2.5 FTE/day. Patients who were identified as using NPWT were reviewed for number of wounds in NPWT, location of dressings, wound bed composition, number of dressing changes, and length of use.

**Results:**

During the 6-month period review, 63 patients were identified as having used NPWT with a total of 239 dressing placements performed by PT, including 113 initial placements and 126 dressing changes. Average dressings per patient was 1.8 (1-12 dressings). Average length of use was 19.2 days (1-87 days). Dressings were placed on all body region with the most frequent placements occurring on the right lower leg (18), L lower arm (13), R lower arm (12), and anterior trunk (11) (Table 1). Wound bed compositions included Full Thickness wounds/subdermal tissues (8), Dermal Substitutes (44), Homograft (5), STSG (52), FTSG (3), Dermal Substitute + STSG (7), and Incisions (2) (Table 2). Three patients had dressings removed early due to bleeding concerns, but no other complications were noted during utilization of NPWT.

**Conclusions:**

PT was able to successful apply a considerable number of NPWT dressings to a variety of wound locations and compositions. A dedicated PT burn team trained in NPWT application allowed for placements utilization anticontracture techniques. PT was also able to maximize range of motion and functional mobility with the dressings in place by considering functional needs throughout application. Patients were not placed on bedrest with NPWT in place nor immobilized. Consistency of PT staff placement also led to improved continuity of care and optimization of outcomes.

**Applicability of Research to Practice:**

Discussion of NPWT placement strategies and practices will benefit less experienced practitioners.

**Funding for the Study:**

N/A